# Comparison of Dynamic Susceptibility Contrast and Arterial Spin Labeling MRI Perfusion in the Assessment of Stroke and Steno-Occlusive Disease: A Systematic Review and Meta-Analysis

**DOI:** 10.3390/diagnostics15131578

**Published:** 2025-06-21

**Authors:** Agnieszka Sabisz, Beata Brzeska, Edyta Szurowska, Arkadiusz Szarmach

**Affiliations:** 2nd Department of Radiology, Medical University of Gdansk, Mariana Smoluchowskiego 17, 80-210 Gdansk, Poland

**Keywords:** dynamic susceptibility contrast, arterial spin labeling, perfusion imaging, brain stroke, steno-occlusive disease

## Abstract

**Background/Objectives**: Assessment of the hemodynamic status of the brain in patients with cerebrovascular diseases is crucial for providing valuable clinical information. Various magnetic resonance perfusion sequences are used in studies, and one of the current challenges is comparing methods utilizing exogenous and endogenous contrast. This meta-analysis aimed to evaluate the correlation between arterial spin labeling (ASL)-derived perfusion parameters and those obtained by dynamic susceptibility contrast (DSC) perfusion in stroke and steno-occlusive diseases. **Methods**: A systematic review and meta-analysis were conducted, including 14 studies that reported correlation coefficients between perfusion MRI sequences in the assessment of stroke or steno-occlusive diseases. The correlation between ASL-derived cerebral blood flow (ASL-CBF) and DSC-derived cerebral blood flow (DSC-CBF) was analyzed, considering different magnetic field strengths (1.5 T and 3.0 T), sequence types, and brain regions. Additionally, real and normalized data were compared. **Results**: A moderate positive correlation was found between ASL-CBF and DSC-CBF (R = 0.464). Subgroup analysis demonstrated that ASL-CBF and DSC-CBF correlated at 3.0 T (R = 0.401) and 1.5 T (R = 0.700). No significant differences were observed in correlation coefficients based on sequence type or brain region. Normalized data demonstrated a higher correlation coefficient compared to real data (Rreal = 0.393, Rnorm = 0.496). Additionally, the correlation coefficient between ASL-CBF and DSC-derived mean transit time (DSC-MTT) for all included studies was R = −0.422. **Conclusions**: ASL-derived perfusion parameters demonstrate moderate-to-high agreement with DSC perfusion parameters in stroke and steno-occlusive patients. These findings support the potential utility of ASL as a non-invasive alternative to DSC perfusion imaging in clinical and research settings.

## 1. Introduction

Despite advances in early diagnosis and available treatment methods, the number of people affected by cerebrovascular diseases—such as acute ischemic stroke, steno-occlusive disease, severe carotid artery disease, or vasospasm—remains significant, owing to the growing and aging population. Magnetic resonance imaging (MRI) perfusion techniques are widely applied to these patients, as it is crucial to assess the brain’s hemodynamic status to provide valuable clinical information to guide patient triage.

With the continuous development of neuroimaging techniques, various magnetic resonance perfusion sequences have been introduced in studies, making it increasingly difficult to compare research results with the existing literature. One of the current challenges is comparing methods that use exogenous and endogenous contrast agents.

Dynamic susceptibility contrast imaging (DSC) is the most widely used MRI perfusion technique for the brain. DSC is a T2* sequence that dynamically captures the signal decrease caused by the injection of a contrast agent, allowing for a quantitative assessment of multiple parameters associated with cerebral hemodynamic changes, such as cerebral blood flow (CBF), cerebral blood volume (CBV), time to peak (TTP), or mean transit time (MTT) [1]. The results of DSC perfusion metrics correlate with single-photon emission computed tomography (SPECT) and positron emission tomography (PET), which are considered the gold standard of cerebral perfusion evaluation [2]. However, the sequence is not without disadvantages. It requires gadolinium contrast administration, which may lead to the deposition of gadolinium compounds [3]. The use of gadolinium contrast agents must be carefully considered for patients with chronic renal insufficiency [4] and is challenging to repeat due to the slow clearance of gadolinium [5].

Arterial spin labeling (ASL) is a non-invasive MRI technique for measuring brain perfusion without the use of contrast agents. ASL uses magnetically labeled water molecules as an endogenous tracer, creating a labeled bolus in the carotid and vertebral arteries [6,7]. Arterial spin labeling enables the measurement of cerebral blood flow through sequences with a single post-labeling delay (PLD) or the assessment of CBF and bolus arrival time using sequences with multiple PLDs.

A key parameter in ASL imaging is arterial transit time (ATT), which significantly influences sequence design, acquisition parameters, and the accuracy of CBF measurements. ATT is defined as the time required for labeled arterial blood to travel from the labeling plane (or the distal edge of the labeling slab) to the imaging voxel [6,8]. This parameter can vary considerably across individuals and brain regions, and is influenced by factors such as age, anatomical location, and pathological conditions, including cerebrovascular disease or stroke. Steno-occlusive disease poses significant challenges for ASL due to increased arterial transit times (ATTs). As a consequence, long delays between labeling and image acquisition are required, leading to a reduced signal-to-noise ratio due to the tracer’s decay rate (T1 relaxation) in the labeled blood.

ASL and DSC perfusion metrics are well-reviewed as valuable diagnostic biomarkers for distinguishing and grading brain neoplasms, such as gliomas [9,10,11]. Also, several meta-analyses comparing these two imaging techniques are prepared for brain tumors [10,12,13] or comparing the accuracy of arterial spin-labeling for localizing the epileptic lesions [14]. Decreased perfusion is a common feature in steno-occlusive diseases and serves as an important marker for stroke diagnostics. The perfusion–diffusion mismatch concept is widely used in MR imaging for acute ischemic stroke [15]. In some cases, patients cannot receive MR contrast agents, making DSC perfusion unavailable; therefore, it is crucial to determine whether similar information can be obtained using a non-contrast perfusion method, such as ASL. Steno-occlusive diseases cause unique acquisition challenges for both DSC and ASL sequences but in different mechanisms. In particular, the extensive network of blood vessels, collaterals, and delayed arrival of blood to the acquisition area may affect the results. While there are so many elements of these two techniques that could influence the results, there is an important aspect to comparing them. Such analysis can help identify goals and pitfalls and determine their potential complementary roles in clinical practice. To the best of our knowledge, there is no meta-analysis comparing the correlation coefficient of ASL and DSC perfusion metrics in steno-occlusive diseases.

Despite recommendations on how to use it, ASL is still not widely used in clinical practice [8]. The availability of various ASL sequences (e.g., pCASL, CASL, PASL, and mTI-ASL) introduces uncertainty when selecting the appropriate method to assess valid perfusion data for different pathologies. Furthermore, ASL presents technical challenges in acquisition and post-processing. This meta-analysis aims to evaluate whether and how ASL-derived perfusion parameters differ from those obtained through DSC perfusion in stroke and steno-occlusive diseases, focusing on studies that directly compare these two methods. Specifically, we aim to determine whether perfusion metrics correlate between techniques and whether they remain comparable across different acquisition and analysis environments (e.g., varying ROIs, field strengths, and acquisition techniques).

## 2. Materials and Methods

### 2.1. Search Procedure

This meta-analysis was performed according to the Preferred Reporting Items for Systematic Reviews and Meta-Analysis (PRISMA 2020) statement [16]. PubMed, Scopus, and Web of Science were searched on 1 March 2025 for studies that reported the comparison of perfusion MRI sequences in the assessment of stroke or steno-occlusive disease. The following search terms were used: ((intravoxel incoherent motion) OR (arterial spin labeling)) AND ((dynamic contrast enhancement) OR (dynamic susceptibility contrast)) AND ((stroke) OR (steno-occlusive disease)). The results were filtered for English and Polish languages only. Detailed search strings are shown in Supplementary Material S1.

### 2.2. Selection Criteria and Data Collection

Firstly, the titles retrieved from three databases were screened to remove duplicates. All subsequent steps were independently performed by two readers (A.S. and B.B.), and any discrepancies were resolved through discussion to reach consensus. Secondly, the remaining studies were reviewed based on their titles and abstracts, using the following exclusion criteria: case reports, systematic reviews and meta-analyses, technical guidelines, animal or phantom studies, and studies on brain tumors or other diseases not selected for review.

Studies were deemed eligible for the meta-analysis if they were original articles involving in vivo MRI conducted on humans using 1.5 T or 3 T scanners, and if they utilized at least two different perfusion methods in patients with stroke or steno-occlusive disease. In the next stage, full-text articles were assessed for eligibility. Records were excluded if they did not contain a direct quantitative comparison of perfusion parameters obtained with different sequences or if they met any of the aforementioned exclusion criteria.

**Figure 1:**
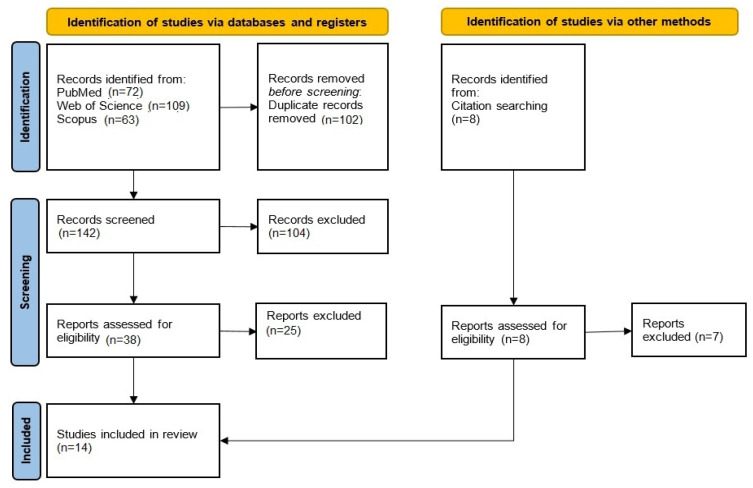
Flow diagram of studies selection based on PRISMA 2020. Flowchart of the study selection process performed in March 2025.

Studies referencing other methods beyond DSC and ASL (such as intravoxel incoherent motion diffusion-weighted imaging) were excluded due to insufficient quantities. Additionally, the reference lists of all included studies were manually reviewed to identify further relevant articles. The step-by-step review process is summarized in Figure 1.

### 2.3. Risk-of-Bias Assessment

The quality assessment of selected studies was performed based on the Quality Assessment of Diagnostic Accuracy Studies (QUADAS-2) tool [17]. Each study was evaluated for potential bias by two independent reviewers (A.S. and B.B.), experienced in perfusion imaging and advanced MRI techniques. Disagreements were resolved by consensus.

### 2.4. Statistical Analysis

For the meta-analysis, data from selected studies were organized into two main comparisons: ASL-CBF versus DSC-CBF, and ASL-CBF versus DSC-MTT. Information extracted by two reviewers from each publication included the following: authors, year of publication, number of patients, age of patients, ASL acquisition techniques, PLD, magnetic field strength, Pearson or Spearman correlation coefficients (R), number and types of ROI territories and ROI method of definition (manual or automatic), and type of values (real or normalized). All DSC post-processing calculation models (such as the DDS curve and SVD algorithm) were considered in the included studies. If an article included both a scan and a rescan after some intervention, only the values prior to the treatment were considered. Any disagreements between the two reviewers were resolved through consensus.

All studies correlating DSC with ASL perfusion parameters used either R or R^2^ values to describe the correspondence. Where necessary, R values were calculated from R^2^ values. The Pearson correlation coefficient (R) was converted to the Spearman correlation coefficient (Rs) using the mathematical formula provided by Rupinski et al. [18]. A random-effects model was used in the meta-analyses to combine the data from the selected studies. Heterogeneity among R values between studies was assessed by calculating the Q statistic and the inconsistency index (I^2^). A *p*-value of <0.05 or an I^2^ value > 50% indicated heterogeneity. To investigate potential sources of heterogeneity, we performed a meta-regression analysis assessing the influence of field strength, type of sequence, normalization or region of interest on CBF ASL and DSC correlation coefficient. We used random-effects meta-regression. Firstly, we prepared the meta-regression with each moderator separately and then with all the moderators. We report the I^2^ and R^2^ to explore possible causes of variability in the data.

In the second step, data for the meta-analysis were grouped and analyzed by field strength (1.5 T and 3 T), types of sequences, ROI territories, and types of values (ROIs with real CBF values versus ROIs with normalized CBF values).

All statistical analyses were performed by one of the reviewers (A.S.), using statistical software TIBCO Software Inc. (Palo Alto, CA, USA, 2017) Statistica version 13.3. A *p*-value of <0.05 was considered statistically significant.

### 2.5. Image Quality

In 8 out of the 29 studies assessed for eligibility, researchers conducted additional qualitative analyses in which two or more independent readers visually inspected maps of perfusion parameters derived from ASL and DSC sequences. Although assessment scales varied across studies, readers generally rated image quality on a scale of poor, weak, moderate, good, or excellent, assigning scores of 1 to 5 points to each map and then averaging the grades from all readers. Two of the studies used only descriptive scales without assigning points or performing statistical analysis, providing only the number of ratings. In one article, only the median and range of results were reported, so this study was excluded from the qualitative analysis. Ultimately, 7 studies assessing image quality were included.

Grading scales across the studies showed slight differences, such as increasing, decreasing, narrower scales (0–3), or purely descriptive formats. To address these discrepancies, min–max normalization was applied in studies where higher ratings corresponded to better quality, and reversed min–max normalization was used in cases where the rating scales were inverted. The normalized results were mapped to a 0–1 scale, where 0 represented the worst image quality, and 1 the best.

## 3. Results

### 3.1. Literature Search

A total of 244 articles were identified through searches in PubMed, Scopus, and Web of Science. After removing 102 duplicate studies, 142 articles remained for further screening. The titles and abstracts of these studies were reviewed, and 104 articles were excluded based on the predetermined exclusion criteria. Subsequently, 38 reports were assessed for eligibility, of which 25 were excluded following a detailed evaluation.

Additionally, eight potentially relevant studies were identified through the reference lists of the selected articles, and one of these was ultimately included. As a result, a total of 14 studies were included in this meta-analysis (Table 1) [19,20,21,22,23,24,25,26,27,28,29,30,31,32].

### 3.2. Bias Assessment

When analyzing the risk of bias, in 3 out of 14 studies, the number of patients was below 15 [19,26,30]. In a few studies, there was a disproportion in gender ratio: 7F/3M [19], 12F/31M [20], 9F/27M [21], 2F/28M [24], and 3F/10M [26]. And in one study, there was no information about the gender of the patients [27]. Two studies were performed on the pediatric cohort [19,29], while the others included only adults. Two studies were found to be from the same research group [28,31]. In three studies [20,23,26] patients were recruited as a part of the prospective imaging study PEGASUS (Perfusion imaging by ASL for clinical use in stroke, WHO International Clinical trials registry no. DRKS00003198), but all of them included patients from different periods of the trial. In six studies [21,22,24,28,29,31], exclusion criteria were not mentioned, but the inclusion criteria were written in detail. In one study, there was no information on inclusion and exclusion criteria [30]. In five studies, ROIs were generated automatically, but in eight studies, ROIs were drawn manually by one observer [19,20,21,22,23,24,25,26]. In one article [20], correction of susceptibility artifacts based on three patients was applied to all patients. In an article prepared by Wolf et al. [21], TTP maps were used to visually define hyper- and hypointense areas, whereas in the article by Wang et al. [28], infarction core was defined as ADC < 550 [×10^−6^ mm^2^/s]. In most of the articles [20,21,23,24,26,27,28,32], ipsilateral hemispheres were normalized to contralateral, but in articles by Goetti et al. [19] and Zhang et al. [25], cerebellum signal was used for normalization, and in Wolf et al. [30], relative CBV values were calculated from the area under the deconvolved tissue concentration curve, and rCBF (regional cerebral blood flow) was measured as the peak value. In Refs. [22,29,31], real values without normalization were used.

### 3.3. Quality Assessment

Seven studies were included in the quality assessment analysis (Table 2). Only in two articles [20,26] were four perfusion maps assessed (ASL-CBF, DSC-CBF, ASL-BAT, and ASL-TTP). In both studies, the ASL sequence type was 3D GRASE mTI-PASL, and DSC maps were graded higher than ASL maps, but in Martin et al. [26], the difference in CBF was not severe. In another two articles [23,33], the pASL technique was used, and the ASL-derived CBF maps were rated very poorly (0.20 and 0.25, respectively). In the three remaining studies, maps from the ASL sequence were equal to maps obtained with the DSC sequence [31,34,35], and in all of them, the pCASL technique was used. Results are summarized in Table 2.

### 3.4. Data Analysis

#### 3.4.1. ASL-CBF vs. DSC-CBF

**Figure 2:**
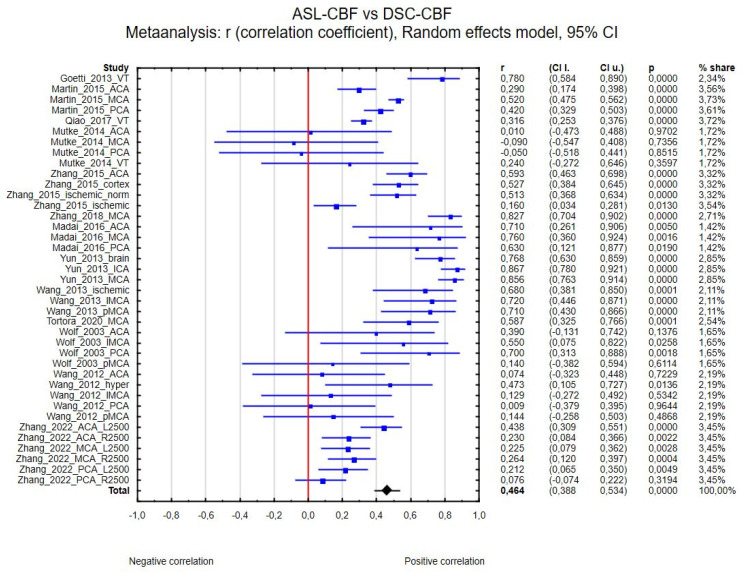
Forest plots of the summary correlation coefficient (r) with corresponding 95% CIs for the correlation between the ASL-CBF values and DSC-CBF values in patients from all eligible studies and regions.

Thirty-nine regions of interest from the 14 studies were included in the ASL-CBF and DSC-CBF comparison. Correlation coefficient values ranged from −0.090 [23] to 0.867 [27]. The correlation coefficient in the meta-analysis between ASL-CBF and DSC-CBF for all included studies was R = 0.464 (95% confidence interval (CI) = 0.388 to 0.534, *p* < 0.001), and the inconsistency index, I^2^, was 87%. Figure 2 presents the forest plot of all eligible studies and regions included in the analysis.

The meta-regression analysis revealed varying levels of heterogeneity across different moderators. When assessing field-strength heterogeneity, the results showed an I^2^ of 89% and an R^2^ of 31%, indicating that this factor explained a significant portion of the observed variability. In contrast, other moderators showed high heterogeneity but failed to explain variation in effect sizes. Specifically, the type of sequence (all) had I^2^ of 87% and R^2^ of 0%, while narrowing the sequence to only pCASL and PASL 3D mTI resulted in I^2^ of 90% and R^2^ of 0%, reinforcing the limited explanatory power of sequence type in this context. Similarly, normalization demonstrated an I^2^ of 87% and an R^2^ of 0%, and the region of interest showed an I^2^ of 82% and an R^2^ of 0%, suggesting that these factors did not significantly contribute to variability. When all moderators were combined in the meta-regression model, the overall I^2^ was 83% (without cases that do not match these moderators), while the R^2^ remained at 0%.

**Figure 3:**
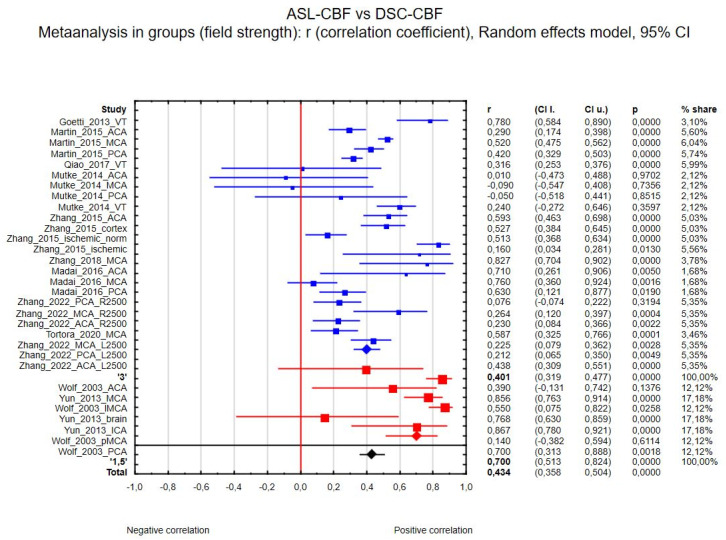
Forest plots of the correlation coefficient (r) subgroups, with corresponding 95% CIs for the correlation between the ASL-CBF values and DSC-CBF values divided by MRI field strength (3 T in blue or 1.5 Tin red, total in black).

**Figure 4:**
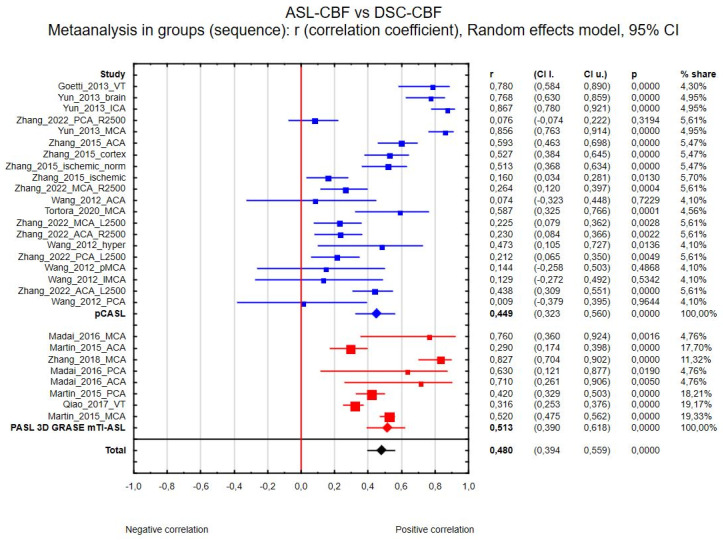
Forest plots of the correlation coefficient (r) subgroups, with corresponding 95% CIs for the correlation between the ASL-CBF values and DSC-CBF values in patients from all eligible studies divided into type of sequence (pCASL in blue, PASL 3D mTI in red, total in black).

**Figure 5:**
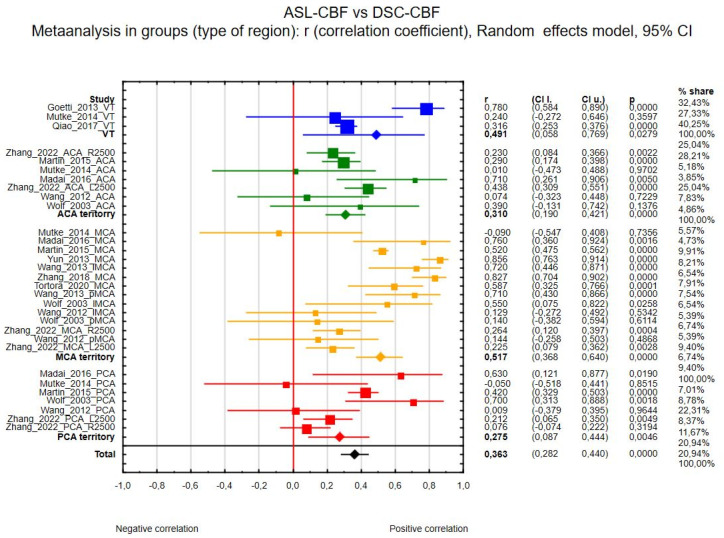
Forest plots of the correlation coefficient (r) subgroups, with corresponding 95% CIs for the correlation between the ASL-CBF values and DSC-CBF values in patients from all eligible studies divided into ACA (in green), MCA (in yellow), PCA (in red), and VT regions (in blue).

**Figure 6:**
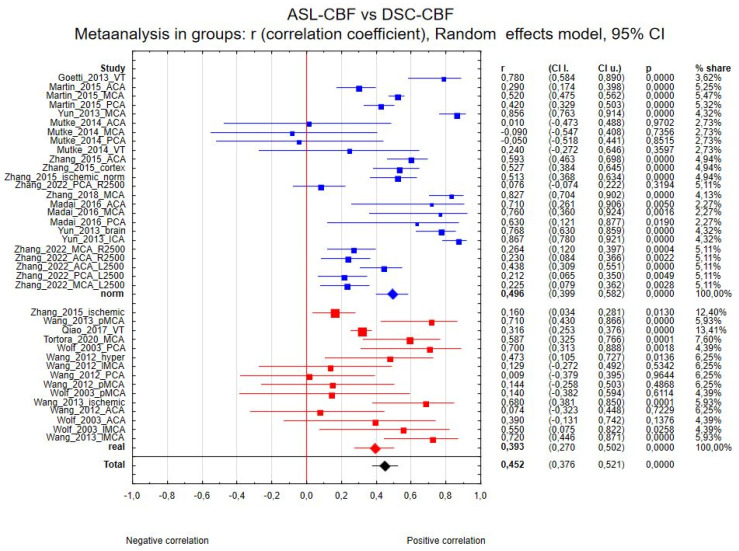
Forest plots of the correlation coefficient (r) subgroups, with corresponding 95% CIs for the correlation between the ASL-CBF values and DSC-CBF values in patients from all eligible studies divided into normalized values (in blue) of perfusion in ROIs or with real values (in red).

The differences in the subgroups were as follows:

(a) Thirty-one regions of interest were included in the ASL-CBF and DSC-CBF comparison between magnetic field strength the correlation coefficients were as follows: for 1.5 T R_1.5_ = 0.700 (7 regions, 95% CI 0.513 to 0.824, *p* < 0.001) and for 3 T R_3.0_ = 0.401 (24 regions, 95% CI 0.319 to 0.477, *p* < 0.001). The between-group effect was Z = −2.75, with *p* = 0.006. Figure 3 presents the forest plot of this analysis.

(b) Twenty-eight regions of interest were included in the ASL-CBF and DSC-CBF comparison between types of sequence; only the analysis between pCASL and PASL 3D GRASE mTI was possible to acquire, because the number of studies with other types of sequences was not sufficient. The correlation coefficients were R_pCASL_ = 0.449 (20 regions, 95% CI 0.323 to 0.560, *p* < 0.001) and R_mTI_ = 0.513 (8 regions, 95% CI 0.390 to 0.618, *p* < 0.001). The between-group effect was Z = 0.759, with *p* = 0.448, so it was not statistically significant. Figure 4 presents the forest plot of this analysis.

(c) Thirty-one regions of interest were included in the ASL-CBF and DSC-CBF comparison between brain regions; the analysis was divided into ACA (anterior cerebral artery), MCA (middle cerebral artery), PCA (posterior cerebral artery) territories, or vessel territories (VTs, including ACA + MCA + PCA and others). The correlation coefficients were as follows: R_VT_ = 0.491 (3 regions, 95% CI 0.058 to 0.769, *p* = 0.028), R_MCA_ = 0.517 (14 regions, 95% CI 0.368 to 0.640, *p* < 0.001), R_PCA_ = 0.275 (7 regions, 95% CI 0.087 to 0.444, *p* = 0.005), and R_ACA_ = 0.310 (7 regions, 95% CI 0.190 to 0.421, *p* < 0.001). There was no significance in the between-group analysis. Figure 5 presents the forest plot of that analysis.

(d) Thirty-nine regions of interest were included in the ASL-CBF and DSC-CBF comparison between types of values. We divided the analysis between ROIs with real CBF values and ROIs with normalized CBF values. The correlation coefficients were R_real_ = 0.393 (15 regions, 95% CI 0.270 to 0.502, *p* < 0.001) and R_norm_ = 0.496 (24 regions, 95% CI 0.399 to 0.582, *p* < 0.001). The between-group effect was Z = 1.374, with *p* = 0.169. Figure 6 presents the forest plot of this analysis.

#### 3.4.2. ASL-CBF vs. DSC-MTT

**Figure 7:**
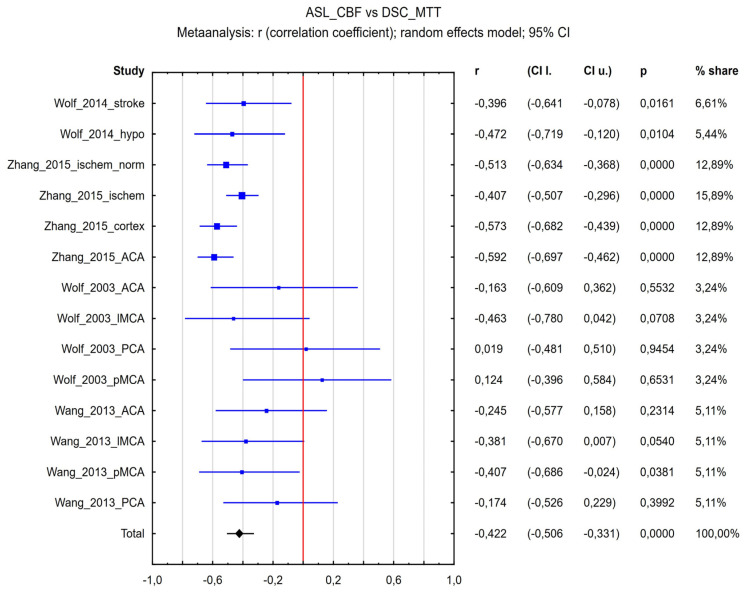
Forest plots of the summary correlation coefficient (r) with corresponding 95% CIs for the correlation between the ASL-CBF values and DSC-MTT values in patients from all eligible studies and regions.

The correlation coefficient between ASL-CBF and DSC-MTT for all included studies (Figure 7) was R = −0.422 (95% confidence interval (CI) = −0.506 to −0.331, *p* < 0.001), and the inconsistency index I^2^ was 42%. Because the value of heterogeneity (I^2^) was low, further analyses were not prepared. Figure 7 presents the forest plot of all eligible studies and regions included in the analysis.

## 4. Discussion

To the best of our knowledge, this is the first meta-analysis of the correlation coefficients between DSC and ASL data in patients with steno-occlusive diseases. Recently, a systematic review was published comparing these techniques in the ischemic penumbra [36], but no one has attempted to analyze them by considering magnetic field strength, type of region of interest, or type of sequence. In our article, we evaluated the correlation coefficients between ASL-CBF and DSC-CBF, as well as ASL-CBF and DSC-MTT, in patients with steno-occlusive diseases and acute ischemic stroke.

The DSC sequence has been widely applied in clinical routines for years and is likely the most frequently used technique for assessing MRI perfusion. It is available on most MRI scanners, and basic post-processing can be performed automatically on any workstation commercially available from all major MRI vendors [37]. Interestingly, a simple test on searching terms “dynamic susceptibility contrast MRI” and “arterial spin labeling MRI” in PubMed (without any filters applied) shows that, in the early 2000s, the number of articles concerning those two types of sequences was nearly equal. However, from 2005 to the present, there have been more articles published on the ASL sequence. When the search results were limited to the year 2021, 106 articles were found for DSC, and 458 for ASL—over four times as many. This means, on average, a new article about ASL is published every day (1.25 articles per day, to be exact).

To summarize the main findings of the meta-analysis, direct comparisons using correlation coefficients were found in 14 studies (53 regions with correlation coefficients included in the analysis), and a visual assessment of the quality of DSC and ASL perfusion maps was performed and reported in detail in seven articles. We identified a moderate positive correlation (0.464) between ASL-CBF and DSC-CBF. Furthermore, we demonstrated that ASL-CBF and DSC-CBF correlate at both 3.0 T and 1.5 T, with a higher correlation coefficient observed for the lower field strength. No differences in correlation coefficients were identified between ASL-CBF and DSC-CBF based on sequence type, analyzed brain regions, or value types.

High correlation was reported in studies conducted on 1.5 T scanners. Moderate correlation was observed in studies on 3 T scanners, also in sequences such as pCASL and PASL 3D GRASE, or CBF values from regions of interest, including the middle cerebral artery and VT, as well as normalized CBF values. Low correlation was found in CBF values derived from the anterior cerebral artery and posterior cerebral artery, along with real CBF values.

Measurements of CBF values by the DSC or ASL technique are influenced in different ways by the presence of the steno-occlusive disease and the presence of collateral vessels. Calamante et al. checked that delay and dispersion in the estimated AIF (Arterial Input Function) in the DSC sequence were found to introduce significant underestimation of cerebral blood flow (CBF) and overestimation of mean transit time (MTT) [38]. Ibaraki et al. presented that in the simulation study and the stroke patient study, underestimation of CBF due to tracer delay was larger for shorter MTTs in the dynamic susceptibility contrast-enhanced MRI [39] and showed that the tracer delay in DSC-MRI causes errors in CBF estimates, even in healthy persons [40]. On the other hand, CBF-ASL calculated by pCASL can be affected by perfusion signal loss because of long transit times in steno-occlusive disease when the labeled blood does not reach the imaging capillary bed in the slice [8], but ASL with multiple delay times can compensate for this effect [41]. Wolf et al. demonstrated whether patients with major transit delay were excluded, and CBF-DSC correlated best with CBF-AS [30]. ASL also presented the focally increased signal intensity, which may be produced by true hyperperfusion or artifacts such as motion or intravascular spin label [42,43]. It might explain the moderate correlation coefficient between CBF-DSC and CBF-ASL. The correlation coefficients may be affected by disease or acquisition factors.

Limitations observed in the included studies mostly showed the problems with ASL sequence. Artifacts in labeled images affect the CBF values. The most often mentioned artifact that disturbs the correlation coefficient between CBF from ASL and DSC or facilitates the proper interpretation of ASL was delayed transit time [27,30]. Yun et al. concluded that the correlation coefficient between CBF measures tends to be weaker when the transit time is more delayed [27]. Following, delayed transit time not only has an impact on CBF calculations on the ASL map but also might lead to arterial transit delay artifacts causing increasing signal in small arteries and hypointense areas where labeled blood has not yet arrived, which is especially common in steno-occlusive patients [20]. Techniques that acquire images at multiple PLDs/TIs are recommended in cases with prolonged or heterogeneous blood transit times, such as steno-occlusive diseases [6,44], because it can collect data points at different stages of the labeled bolus arrival, allowing for a more accurate estimation of the delivered signal [45]. Furthermore, susceptibility effects can indeed cause issues in ASL imaging, primarily leading to signal dropout and distortion, and also potentially affecting labeling efficiency. Furthermore, correction of susceptibility distortions in ASL improves the diagnostic performance and increases the correlation coefficient [20,26]. Last but not least, ASL sequences do not allow for the quantification of CBF in white matter [24]. The white matter perfusion is difficult to acquire due to low SNR caused by the lower blood flow and longer ATT compared to grey matter [8]. If the post-labeling delay (PLD) used in the ASL sequence is shorter than the ATT, the labeled blood may not reach the capillary bed in the white matter at the time of imaging, leading to an underestimation of CBF or artefactual low signal. Iutaka et al. provided a brief summary of the ASL potential pitfalls and artifacts and provided a practical approach for interpreting ASL images [46].

Zhang et al. explained the differences between the correlation coefficient between studies in the analysis of CBF parameters, among others, to be differences in ASL acquisition techniques [25]. Our meta-analysis compared only two types of ASL sequences—pCASL and 3D mTI GRASE (gradient and spin echo)—but we do not support Zhang’s conclusion. Three-dimensional GRASE may be a promising sequence for steno-occlusive disease because it was shown that it could correct for the delayed arrival time due to the use of multiple inflow times [20,47]. Further studies should be prepared to prove the potential.

For relative comparison and interpretation, especially in the context of localized disease or age-related changes, normalization often involves adjusting regional CBF to a global value or comparing it to a value from a presumed healthy reference region, like the contralateral hemisphere or a specific unaffected territory [8]. In our study, normalization was most commonly performed using the region on the contralateral side or a cerebellar area. Zhang et al. showed that, after normalization, the correlation coefficients were higher between ASL values and DSC parameters than for absolute values [24]. After that, Zhang et al. also concluded that normalization by values in the cerebellum helps to standardize the values of reference [25]. In our meta-analysis, we showed that normalization of CBF values is worthwhile because of the higher correlation between ASL and “gold standard” DSC MRI. We could not prepare a meta-analysis divided by the type of normalization. Further analysis is needed to check Zhang’s agreement.

Moreover, ROIs can be defined based on anatomical landmarks or specific brain regions. For cerebrovascular disease evaluation, scoring systems like ASPECTS can be used for regional analysis, delineating cortical perfusion territories, but for Moyamoya disease, Fan et al. assumed that it should be different [44]. The appropriate ROI size can vary depending on the goal and the size of the expected abnormality [44]. The method of generation of region on interest (manual or automatic) could introduce the variability—by differences in ROI size, location, and delineation criteria. In Table 1, we present information about the choice of the ROI. We would like to emphasize the need for automatic standardized ROI or normalization frameworks, such as MNI space templates or vascular territory-based atlases [48,49], to enhance reproducibility and comparability across studies

Our results show that CBF on 1.5 T field strength has a higher correlation between ASL and DSC sequence than on 3 T. The high value results mainly from Yun et al.’s study [27]. Yun et al. presented a very high correlation (from 0.768 to 0.867) between ASL-CBF and DSC-CBF in patients with moyamoya disease. Moyamoya patients demonstrate irregular collaterals which influence the high delay times. So, Yun et al.’s results using the 1.5 T scanner are challenging to explain. ASL acquisition on higher-field-strength scanners should be more precise and reliable because the higher the field is, the higher the signal-to-noise and contrast-to-noise ratios we obtain [50]. Also, longer T1 relaxation times on 3 T scanners benefit ASL sequences [8]. In our opinion, the high correlation observed in this subgroup may be influenced by sample characteristics and study-specific factors, including disease-related hemodynamic patterns and methodological consistency within the cohort. Our meta-regression findings highlight the impact of field strength as the source of heterogeneity as a key contributor of variability in this analysis. Future studies should further investigate ASL performance across different field strengths using larger and more diverse populations to validate these preliminary findings.

The analysis of CBF map quality revealed that DSC either has higher quality or is comparable to the ASL sequence. In most of the studies, both DSC and ASL techniques presented good quality. However, due to the limited number of studies, we were unable to conduct a group meta-analysis based on magnetic field strength or sequence type. Most of the studies that we considered in the quality assessment were conducted on 3 T scanners, and the quality varied between techniques. Among these, Wang et al. showed that image quality was significantly higher for both ASL and DSC images acquired at 3 T versus 1.5 T in hypoperfusion lesions [31]. A low signal-to-noise (SNR) ratio is a known limitation of ASL [6]. Additionally, the type of acquisition technique in ASL can vary significantly, necessitating further investigation. However, it can still benefit certain patients for whom gadolinium contrast may be contraindicated.

The moderate correlation contributes to ASL currently holding a reserve role in the evaluation of CBF-ASL compared to DSC. This reserve role is particularly important and beneficial when gadolinium contrast agents are contraindicated for DSC, such as in patients with impaired renal function. ASL can provide valuable perfusion information similar to DSC in many cases; its limitations in regard to sensitivity, especially for small lesions, or in regions like white matter, mean it may not be equivalent to DSC in all clinical contexts. These constraints significantly reduce its potential as a standalone alternative to DSC, as certain pathological changes may go undetected or be less accurately assessed using ASL alone. The moderate correlation highlights that while ASL can provide valuable non-contrast perfusion information useful in specific clinical situations, it is not a perfect substitute for DSC, particularly when assessing subtle abnormalities or evaluating regions like white matter. Furthermore, a considerable number of the ASL sequences referenced in this study are not readily available in routine clinical practice. The lack of widespread accessibility and standardization of these sequences poses additional challenges for integrating ASL findings into everyday clinical workflows, ultimately hindering the immediate applicability of the results. Consequently, further research and technological advancements are necessary to address these limitations and improve the feasibility of ASL for broader clinical adoption.

Our study has several limitations. The main drawback of this meta-analysis is the number of studies included. Although 142 studies were screened, most of them were unsuitable for inclusion in the meta-analysis, primarily due to the lack of direct comparisons between the ASL and DSC sequences using correlation coefficients. Due to the limited number of studies available for inclusion, the meta-analysis was constrained to examine only two ASL sequence types: pCASL and PASL mTI. While these types of sequences are the most common of ASL acquisition techniques, they may not fully capture potential variations in performance across different ASL implementations. ASL imaging is highly technique-dependent, with significant variability in labeling approaches (e.g., pulsed ASL and pseudo-continuous ASL); readout methods (e.g., 2D vs. 3D acquisitions); and post-processing steps, such as normalization and quantification strategies. These methodological differences can influence the reported correlations between ASL and DSC, impacting the generalizability of our findings. For the same reason, a meta-analysis for different normalization types could not be performed.

Additional inaccuracies may have arisen due to variability in acquisition parameters across studies—not only between different ASL sequence types but also within DSC perfusion. Furthermore, variations in analysis and post-processing steps might have introduced bias, particularly concerning differences in ROI placement strategies, such as manually drawn ROIs versus those automatically derived from vascular brain territory atlases.

Several subgroup analyses—such as PASL vs. pCASL and 1.5 T vs. 3 T—were based on a relatively small number of studies (typically 4–5). However, it is important to note that these analyses were conducted using values extracted from regions of interest reported in the included articles. As a result, the amount of data considered in each analysis was substantially larger than the number of studies alone might suggest. The small number of correlation coefficients in ROIs in certain subgroups limits the reliability of the findings, warranting cautious interpretation of the results. A small pool of studies limits the ability to explore potential confounding factors comprehensively, potentially leading to biased or less generalizable results. It is crucial to interpret the results with caution, recognizing that findings derived from subgroups with fewer studies may not fully capture the agreement between ASL and DSC sequence. Future research should prioritize expanding the evidence base by incorporating additional studies and exploring subgroup effects with more extensive datasets.

Moreover, not all included acquisition techniques of the ASL sequences used in the studies are commercially available. Many are not included in standard clinical libraries and are distributed as research-only sequences. This limitation may make it challenging to implement the conclusions of this meta-analysis in clinical practice.

## 5. Conclusions

In conclusion, this meta-analysis provides a comprehensive evaluation of the correlation coefficients between DSC and ASL MRI techniques in patients with steno-occlusive diseases and acute ischemic stroke. Our findings underscore a moderate positive correlation between ASL-CBF and DSC-CBF, highlighting differences based on magnetic field strength and acquisition parameters. While DSC remains a well-established technique widely applied in clinical routines, ASL offers significant potential, especially for patients where gadolinium contrast is contraindicated.

Several challenges, such as delayed transit time and artifacts in ASL sequences, were identified as factors affecting correlation coefficients and diagnostic reliability. Nevertheless, the use of advanced ASL acquisition techniques and normalization methods can enhance its comparability with the “gold standard” DSC approach. Our results emphasize the importance of continued research to refine these techniques and explore their complementary roles in clinical practice. Future studies should address current limitations, particularly in white matter perfusion and sequence standardization, to improve diagnostic accuracy and patient outcomes.

## Figures and Tables

**Table 1:** Summary of articles involved in the meta-analysis with data used to correlate DSC and ASL sequences.

	Patients	Field Strength (T)	ASL Type	PLD or mTI (ms)	Number of Patients	Number of ROIs Analyzed	Correlation Type (P—Pearson; S—Spearman)	r	Perfusion Parameters		Region (m—Manual; a—Automatic)	Normalization with Reference Zone (1) or Real Values (0)	Ref.
									DSC	ASL			
Goetti 2013	Moyamoya disease	3.0	pCASL	1500	10	30	P	0.79	CBF	CBF	ACA + MCA + PCA (m)	1 (cerebellum)	[19]
Martin 2015	Steno-occlusive disease	3.0	3D GRASE mTI-PASL	15× TI, 300–3100, 200 increment	43	258	S	0.29	CBF	CBF	ACA (m)	1 (contralateral)	[20]
						1075	S	0.52	CBF	CBF	MCA (m)	1 (contralateral)	
						344	S	0.42	CBF	CBF	PCA (m)	1 (contralateral)	
Wolf 2014	Ischemic stroke	1.5	3D GRASE mTI-PASL	10× TI, 250–2500,	36	36	P	−0.41	MTT	CBF	Ischemic hemisphere (m)	1 (contralateral)	[21]
						28	P	−0.49	MTT	CBF	Hypoperfusion ROI (m)	1 (contralateral)	
Qiao 2017	Moyamoya disease	3.0	3D GRASE mTI-PASL	16× TI, 480–4080,	41	824	S	0.32	CBF	CBF	MCA + ACA (m)	0	[22]
Mutke 2014	Steno-occlusive disease	3.0	PASL	1800	17	17	S	0.24	CBF	CBF	ACA + MCA + PCA (m)	1 (contralateral)	[23]
						17	S	0.01	CBF	CBF	ACA territory GM (m)	1 (contralateral)	
						17	S	−0.09	CBF	CBF	MCA territory GM (m)	1 (contralateral)	
						17	S	−0.05	CBF	CBF	PCA territory GM (m)	1 (contralateral)	
Zhang 2015	Ischemic stroke	3.0	pCASL	1525	30	120	P	0.53	CBF	CBF	Ischemic hemisphere (m)	1 (contralateral)	[24]
						240	P	0.17	CBF	CBF	Ischemic hemisphere (m)	0	
						120	P	0.55	CBF	CBF	Cortex (m)	1 (contralateral)	
						120	P	0.611	CBF	CBF	Anterior circulation (m)	1 (contralateral)	
						120	P	−0.53	MTT	CBF	Ischemic hemisphere (m)	1 (contralateral)	
						240	P	−0.42	MTT	CBF	Ischemic hemisphere (m)	0	
						120	P	−0.59	MTT	CBF	Cortex (m)	1 (contralateral)	
						120	P	−0.61	MTT	CBF	Anterior circulation (m)	1 (contralateral)	
Zhang 2018	Moyamoya disease	3.0	3D GRASE mTI-PASL	16× TI, 480–4080,	45	45	P	0.84	CBF	CBF	Lateral MCA territories and basal ganglia (m)	1 (cerebellum)	[25]
Madai 2016	Steno-occlusive disease	3.0	3D GRASE mTI-PASL	15× TI, 300–3100, 200 increment	13	13	S	−0.22	CBF	CBF	ACA (a)	1 (contralateral)	[26]
						13	S	0.58	CBF	CBF	MCA (a)	1 (contralateral)	
						13	S	0.58	CBF	CBF	PCA (a)	1 (contralateral)	
Yun 2013	Moyamoya disease	1.5	pCASL	1500	54	54	P	0.87	CBF	CBF	MCA (a)	1 (cerebellum)	[27]
						54	P	0.88	CBF	CBF	ICA (a)	1 (cerebellum)	
						54	P	0.78	CBF	CBF	Anatomical structure-based ROIs (a)	1 (cerebellum)	
Wang 2013	Acute stroke of MCA	1.5/3.0	3D GRASE mTI-pCASL	4× PLD, 1500–3000, 500 increment	24	24	P	0.74	CBF	CBF	Leptomeningeal MCA (a)	0	[28]
						24	P	0.73	CBF	CBF	Perforator MCA (a)	0	
						24	P	0.7	CBF	CBF	Infarct ROI (a)	0	
Tortora 2020	Moyamoya disease	3.0	pCASL	2000	37	37	S	0.59	CBF	CBF	MCA (a)	0	[29]
Wolf 2003	Acute and/or chronic cerebrovascular disease	1.5	CASL	1500	8	16	P	0.41	CBF	CBF	ACA (a)	0	[30]
						16	P	0.57	CBF	CBF	Leptomeningeal MCA (a)	0	
						16	P	0.72	CBF	CBF	PCA (a)	0	
						16	P	0.78	CBF	CBF	Perforator MCA (a)	0	
						16	P	−0.17	MTT	CBF	ACA (a)	0	
						16	P	−0.48	MTT	CBF	Leptomeningeal MCA (a)	0	
						16	P	0.02	MTT	CBF	PCA (a)	0	
						16	P	0.13	MTT	CBF	Perforator MCA (a)	0	
Wang 2012	Acute ischemic stroke	1.5/3.0	pCASL	2000	26	26	S	0.07	CBF	CBF	ACA (a)	0	[31]
						26	S	0.13	CBF	CBF	Leptomeningeal MCA (a)	0	
						26	S	0.14	CBF	CBF	Perforator MCA (a)	0	
						26	S	0.01	CBF	CBF	PCA (a)	0	
						26	S	0.47	CBF	CBF	Hypoperfusion lesion (a)	0	
						26	S	−0.24	MTT	CBF	ACA (a)	0	
						26	S	−0.38	MTT	CBF	Leptomeningeal MCA (a)	0	
						26	S	−0.41	MTT	CBF	Perforator MCA (a)	0	
						26	S	−0.17	MTT	CBF	PCA (a)	0	
Zhang 2022	Moyamoya disease	3.0	pCASL	2500	174	174	S	0.438	CBF	CBF	ACA (left) (a)	1 (cerebellum)	[32]
						174	S	0.23	CBF	CBF	ACA (right) (a)	1 (cerebellum)	
						174	S	0.225	CBF	CBF	MCA (left) (a)	1 (cerebellum)	
						174	S	0.264	CBF	CBF	MCA (right) (a)	1 (cerebellum)	
						174	S	0.212	CBF	CBF	PCA (left) (a)	1 (cerebellum)	
						174	S	0.076	CBF	CBF	PCA (right) (a)	1 (cerebellum)	

**Table 2:** Summary of articles included in the qualitative analysis of quality assessment.

	Patients	Field Strength (T)	ASL Type	Number of Patients	Original Scale	Original Results	Normalized Results (Values Between 0 and 1: 0—Poor Quality; 1—Excellent Quality)				Reference
							ASL-CBF	DSC-CBF	ASL-BAT	DSC-TTP	
Martin 2015	Steno-occlusive disease	3.0	3D GRASE mTI-PASL	43	1—very good 2—good 3—sufficient 4—uninterpretable	Mean score ASL-CBF: 2.0 DSC-CBF: 1.8 ASL-BAT: 3.0 DSC-TTP: 2.1	0.667	0.733	0.333	0.633	[20]
Huck 2012	Subacute ischemia	3.0	pASL	15	0—poor 1—weak 2—moderate 3—good 4—excellent	Mean score ASL-CBF: 0.8 DSC-TTP: 2.53	0.200	-	-	0.633	[33]
Mutke 2014	Steno-occlusive disease	3.0	pASL	28	Good Medium Sufficient Uninterpretable	Number of each answer good/medium/sufficient/uninterpretable ASL-CBF 0/4/13/11 DSC-CBF 13/15/0/0 DSC-TTP 13/12/2/1	0.250	0.821	-	0.774	[23]
Madai 2016	Steno-occlusive disease	3.0	3D GRASE mTI-PASL	13	1—very good 2—good 3—sufficient 4—uninterpretable	mean score ASL-BAT—2.8 DSC-TTP—1.9 ASL-CBF—2.7 DSC-CBF—2.0	0.433	0.667	0.400	0.700	[26]
Hernandez 2012	Acute ischemic stroke	3.0	pCASL	28	Good to excellent Fair Poor Uninterpretable	Percent of each answer Good to excellent/fair/poor/uninterpretable ASL-CBF: 82.1%/7.1%/10.7%/0% DSC-TTP: 78.6%/7.1%/14.3%/0%	0.905	-	-	0.881	[34]
Wang 2012	Acute ischemic stroke	1.5/3.0	pCASL	26	0–3	Mean score ASL-CBF—2.44 DSC-CBF—2.39	0.813	0.797	-	-	[31]
Bokkers 2012	Acute ischemic stroke	3.0	pCASL	105	Good Fair Poor Uninterpretable	Number of each answer good/fair/poor/uninterpretable DSC 75/15/10/5 ASL 76/17/9/3	0.860	0.841	-	-	[35]

## Data Availability

The datasets used and analyzed during the current study are available from the corresponding author upon reasonable request.

## Supplementary Materials

The following supporting information can be downloaded at: https://www.mdpi.com/article/10.3390/diagnostics15131578/s1, Supplementary_search_history S1.

## Abbreviations

ASL	arterial spin labeling
DSC	dynamic susceptibility contrast
MRI	magnetic resonance imaging
MTT	mean transit time
CBF	cerebral blood flow
rCBF	regional cerebral blood flow
CBV	cerebral blood volume
TTP	time to peak
SPECT	single-photon emission computed tomography
PET	positron emission tomography
PLD	post-labeling delay
ATT	arterial transit time
3D-GRASE	three-dimensional gradient and spin echo
pCASL	pseudo-continuous arterial spin labeling
CASL	continuous arterial spin labeling
PASL	pulsed arterial spin labeling
mTI-ASL	multi-time inversion arterial spin labeling
ROI	region of interest
PRISMA	Preferred Reporting Items for Systematic Reviews and Meta-Analyses
QUADAS	Quality Assessment of Diagnostic Accuracy Studies
ASL-BAT	arterial spin labeling–bolus arrival time
ACA	anterior cerebral artery
MCA	middle cerebral artery
PCA	posterior cerebral artery
SNR	signal-to-noise ratio
