# Supplementary material for: Comparison of Dynamic Susceptibility Contrast and Arterial Spin Labeling MRI Perfusion in the Assessment of Stroke and Steno-Occlusive Disease: A Systematic Review and Meta-Analysis
Source: Diagnostics (Basel). 2025 Jun 21;15(13):1578. doi: 10.3390/diagnostics15131578 (PMC12249046; doi:10.3390/diagnostics15131578)
Supplement: Supplementary file 1 [file diagnostics-15-01578-s001.zip › diagnostics-3589858-supplementary.pdf]

PubMed

(("intravoxel"[All Fields] AND ("incoherence"[All Fields] OR "incoherences"[All Fields] OR "incoherency"[All Fields] OR "incoherent"[All Fields] OR "incoherently"[All Fields]) AND ("motion"[MeSH Terms] OR "motion"[All Fields] OR "motions"[All Fields] OR "motion s"[All Fields] OR "motional"[All Fields] OR "motionally"[All Fields])) OR (("arterialization"[All Fields] OR "arterializations"[All Fields] OR "arterialize"[All Fields] OR "arterialized"[All Fields] OR "arterializing"[All Fields] OR "arterially"[All Fields] OR "arterials"[All Fields] OR "arterie"[All Fields] OR "arteries"[MeSH Terms] OR "arteries"[All Fields] OR "arterial"[All Fields] OR "arteris"[All Fields] OR "artery"[All Fields] OR "arterious"[All Fields] OR "artery s"[All Fields] OR "arterys"[All Fields]) AND "spin"[All Fields] AND ("label"[All Fields] OR "label s"[All Fields] OR "labeled"[All Fields] OR "labeler"[All Fields] OR "labelers"[All Fields] OR "labelings"[All Fields] OR "labelled"[All Fields] OR "labeller"[All Fields] OR "labellers"[All Fields] OR "labellings"[All Fields] OR "labels"[All Fields] OR "product labeling"[MeSH Terms] OR ("product"[All Fields] AND "labeling"[All Fields]) OR "product labeling"[All Fields] OR "labeling"[All Fields] OR "labelling"[All Fields]))) AND ((("dynamer"[All Fields] OR "dynamers"[All Fields] OR "dynamic"[All Fields] OR "dynamical"[All Fields] OR "dynamically"[All Fields] OR "dynamicity"[All Fields] OR "dynamics"[All Fields] OR "dynamism"[All Fields] OR "dynamisms"[All Fields]) AND ("contrast media"[Pharmacological Action] OR "contrast media"[MeSH Terms] OR ("contrast"[All Fields] AND "media"[All Fields]) OR "contrast media"[All Fields] OR "contrast"[All Fields] OR "contrasted"[All Fields] OR "contrasting"[All Fields] OR "contrastive"[All Fields] OR "contrastively"[All Fields] OR "contrastiveness"[All Fields] OR "contrastivity"[All Fields] OR "contrasts"[All Fields]) AND ("enhance"[All Fields] OR "enhanced"[All Fields] OR "enhancement"[All Fields] OR "enhancements"[All Fields] OR "enhancer"[All Fields] OR "enhancer s"[All Fields] OR "enhancers"[All Fields] OR "enhances"[All Fields] OR "enhancing"[All Fields])) OR (("dynamer"[All Fields] OR "dynamers"[All Fields] OR "dynamic"[All Fields] OR "dynamical"[All Fields] OR "dynamically"[All Fields] OR "dynamicity"[All Fields] OR "dynamics"[All Fields] OR "dynamism"[All Fields] OR "dynamisms"[All Fields]) AND ("disease susceptibility"[MeSH Terms] OR ("disease"[All Fields] AND "susceptibility"[All Fields]) OR "disease susceptibility"[All Fields] OR "susceptibilities"[All Fields] OR "susceptibility"[All Fields] OR "susceptible"[All Fields] OR "susceptibles"[All Fields] OR "susceptive"[All Fields] OR "susceptivity"[All Fields]) AND ("contrast media"[Pharmacological Action] OR "contrast media"[MeSH Terms] OR ("contrast"[All Fields] AND "media"[All Fields]) OR "contrast media"[All Fields] OR "contrast"[All Fields] OR "contrasted"[All Fields] OR "contrasting"[All Fields] OR "contrastive"[All Fields] OR "contrastively"[All Fields] OR "contrastiveness"[All Fields] OR "contrastivity"[All Fields] OR "contrasts"[All Fields]))) AND ("stroke"[MeSH Terms] OR "stroke"[All Fields] OR "strokes"[All Fields] OR "stroke s"[All Fields] OR ("steno-

occlusive"[All Fields] AND ("disease"[MeSH Terms] OR "disease"[All Fields] OR "diseases"[All Fields] OR "disease s"[All Fields] OR "diseased"[All Fields])))

Filters: English and Polish language only
Date of search: 1 March 2025

Scopus

TITLE-ABS-KEY ((((intravoxel AND incoherent AND motion) OR (arterial AND spin AND labeling)) AND ((dynamic AND contrast AND enhancement) OR (dynamic AND susceptibility AND contrast)) AND ((stroke) OR (steno-occlusivel AND disease))))

Filters: English and Polish language only
Date of search: 1 March 2025

Web of Science

All fields
(((intravoxel incoherent motion) OR (arterial spin labeling)) AND ((dynamic contrast enhancement) OR (dynamic susceptibility contrast)) AND ((stroke) OR (steno-occlusive disease)))

Filters: English and Polish language only
Date of search: 1 March 2025
